# Corrigendum to: Metal tolerance protein MTP6 affects mitochondrial iron and manganese homeostasis in cucumber

**DOI:** 10.1093/jxb/ery399

**Published:** 2018-11-27

**Authors:** Magdalena Migocka, Ewa Maciaszczyk-Dziubinska, Karolina Małas, Ewelina Posyniak, Arnold Garbiec

**Affiliations:** 1 University of Wroclaw, Institute of Experimental Biology, Department of Plant Molecular Physiology, Kanonia, Wroclaw, Poland; 2 University of Wroclaw, Institute of Experimental Biology, Department of Genetics and Cell Physiology, Kanonia, Wroclaw, Poland; 3 University of Wroclaw, Institute of Experimental Biology, Department of Animal Developmental Biology, Sienkiewicza, Wroclaw, Poland

*Journal of Experimental Botany*, Advance Access publication: 9 October 2018, doi:10.1093/jxb/ery342

The online version of this article mislabelled the protein, MTP6, in the title, as ‘MPT6’. This error has now been corrected.

